# Prevalence and occupational exposure to zoonotic diseases in high-risk populations in the Free State Province, South Africa

**DOI:** 10.3389/fmicb.2023.1196044

**Published:** 2023-06-05

**Authors:** Cornelius G. van der Westhuizen, Felicity J. Burt, Nina van Heerden, Willie van Zyl, Tonia Anthonissen, Jolly Musoke

**Affiliations:** ^1^Department of Medical Microbiology, Faculty of Health Sciences, University of the Free State, Bloemfontein, South Africa; ^2^Division of Virology, National Health Laboratory Service, Universitas, Bloemfontein, South Africa; ^3^Division of Virology, Faculty of Health Sciences, University of the Free State, Bloemfontein, South Africa; ^4^Department of Agriculture, Land Reform and Rural Development, Division of Veterinary Services, Kroonstad, South Africa; ^5^Department of Medical Microbiology, National Health Laboratory Service, Universitas, Bloemfontein, South Africa

**Keywords:** bovine TB transmission, hantavirus, *Leptospira*, zoonotic, risk factors, brucellosis, seroprevalence, tuberculosis

## Abstract

**Introduction:**

Zoonotic diseases are responsible for 2.5 billion human cases globally and approximately 2.7 million deaths annually. Surveillance of animal handlers and livestock for zoonotic pathogens contributes to understanding the true disease burden and risk factors within a community. This study investigated the prevalence of selected zoonoses in cattle, farm workers and occupational exposure to endemic zoonotic diseases and their associated risk factors.

**Methods:**

Sputum samples from farmworkers were screened for *Mycobacterium bovis*. Blood specimens from farmworkers and archived sera were tested for serological evidence of *Brucella* sp., hantaviruses, and *Leptospira* sp. Communal and commercial cattle herds were tested for bovine tuberculosis and brucellosis.

**Results:**

*Mycobacterium bovis* was not isolated from human samples. A total of 327 human sera were screened, and 35/327 (10.7%) were *Brucella* sp. IgG positive, 17/327 (5.2%) *Leptospira* sp. IgM positive, and 38/327 (11.6%) hantavirus IgG positive (95% CI). A higher proportion of *Brucella* sp. IgG-positive samples were detected among veterinarians (value of *p* = 0.0006). Additionally, two cattle from a commercial dairy farm were bovine tuberculosis (bTB) positive using the bTB skin test and confirmatory interferon-gamma assay. A higher percentage of confirmed brucellosis-positive animals were from communal herds (8.7%) compared to commercial herds (1.1%).

**Discussion:**

These findings highlight the brucellosis and *M. bovis* prevalence in commercial and communal herds, the zoonotic disease risk in commercial and subsistence farming in developing countries, and the occupational and rural exposure risk to zoonotic pathogens.

## 1. Introduction

Zoonoses are transmitted from vertebrate animals to humans and are accountable for more than 60% of all recognized human diseases and 75% of emerging infectious diseases (EID; Jones et al., 2008). In developing countries, including South Africa (SA), the mortality rate associated with EID is 47.3% (Wang et al., 2016). The majority (71.8%) of EID originate from wildlife (Jones et al., 2008). The pandemic potential of EID, as seen in the recent severe acute respiratory syndrome coronavirus 2 (SARS-CoV-2) outbreak, justifies increased preparedness and research to understand these pathogens (LeDuc and Barry, 2004). Low- and middle-income countries are potentially more at risk of zoonotic pathogenic outbreaks due to limited resources. Hence, surveillance studies are vital to determine disease burden with potential public and veterinary health implications.

*Mycobacterium bovis* is the etiological agent for bovine tuberculosis (bTB) in cattle and zoonotic tuberculosis (TB) in humans. Evidence suggests an increase in intra- and inter-species transmission of *M. bovis* has previously been reported in South Africa, with bTB infection confirmed in cattle and 16 different animal species, including one domestic porcine and 15 wildlife species (Hlokwe et al., 2014). However, the prevalence of human TB due to *M. bovis* remains relatively unknown in South Africa and globally, primarily due to routine diagnostics being unable to differentiate between *M. bovis* and *Mycobacterium tuberculosis*. Though the two *Mycobacterium* species are genetically closely related, *M. bovis* does require a different treatment as it is inherently resistant to pyrazinamide, an important first-line medication used in a TB drug regimen (World Health Organization, 2017). A crude estimated rate of overall median proportions of zoonotic TB incidents was 2.8% for African countries (Müller et al., 2013). In South Africa, bTB and bovine brucellosis (BB) infection in cattle are controlled diseases associated with extensive morbidity that consequently lead to livestock production losses. Furthermore, human diseases caused by these bacteria are a notifiable condition due to their associated mortality and morbidity and, therefore, a considerable public health concern with substantial economic impact.

*Brucella* spp. are the etiological agents for brucellosis, commonly referred to as undulant fever or Malta fever. *Brucella abortus*, responsible for BB, is considered one of the predominant zoonotic pathogens in animals and humans (Rahman et al., 2017). Brucellosis is annually responsible for approximately 500 000 new human cases worldwide, with most cases reported in regions where the disease has reached levels of endemicity (Lai et al., 2017). The last formally published study focusing on the incidence rate of *Brucella* sp. in the South Africa human population reported a rate of >0.2 per 100 000 population based on a survey from 1956 to 1959 (Schrire, 1962). Sporadic cases have been reported subsequently, but surveillance remains limited (Govindasamy, 2020).

Leptospirosis, also known as Weil’s disease, is a widespread and potentially fatal zoonotic bacterial disease transmitted to humans from contact with infected animals’ urine (Haake and Levett, 2015). Globally, between 300,000 and 500,000 cases of leptospirosis are reported each year, with case fatality rates of up to 30% (Tilahun et al., 2013). In South Africa, an annual incidence of between 0.15 and 0.66/100,000 population has been documented, with sporadic outbreaks reported (Naidoo et al., 2020; Gizamba et al., 2022).

Hantaviruses are transmitted to humans from contact with excreta from infected rodents and have been identified as the cause of mild to severe diseases with fatalities in most parts of the world except Africa. Serological evidence of hantaviruses has been detected in sub-Saharan, East-, and West Africa, and molecular evidence in rodents has been described. However, there are no reports on human disease except occasionally imported cases (Moolla et al., 2022). The limited studies on potential rodent and insectivore hosts have not, to date, shown evidence of hantavirus infection in hosts in South Africa, although limited surveillance studies in humans have suggested a low level of serological evidence. The moderately low seroprevalence rate from patients in South Africa does not exclude the possibility of hantavirus disease occurring in the country and certainly justifies further investigation.

Zoonotic pathogen surveillance studies at the animal-human interface and among populations at occupational risk with direct animal exposure or exposure due to residing in rural conditions are crucial for identifying circulating pathogens with public health implications. These zoonoses clinically present with symptoms generally shared with a range of other common infectious diseases (i.e., malaria, typhoid fever), leading to difficulties in diagnosis and underestimating the true burden of these diseases (Salyer et al., 2017).

This study aimed to investigate the prevalence and associated risk factors of *M. bovis* and *Brucella* sp. in cattle and farmworkers from two farming communities: communal or backyard (subsistence) farming and large-scale commercial farming. Furthermore, this study aimed to document occupational and environmental exposure to *Brucella* sp., *Leptospira* sp., and hantaviruses across the Free State province, South Africa.

## 2. Materials and methods

### 2.1. Study design

A prospective, cross-sectional study was conducted using two populations which included workers with occupational exposure to animals and cattle. The study was conducted between November 2019 and March 2020. All farms selected for this study had cattle scheduled for routine bTB and BB screening as part of the Department of Agriculture Land Reform and Rural Development (DALRRD) Free State province surveillance program. Subsequently, farmworkers on the chosen farms were screened for *Mycobacterium tuberculosis* complex (MTBC) and *Brucella* IgG antibodies and asked to complete a questionnaire.

Furthermore, a retrospective analysis was performed on archived serum samples from workers with occupational exposure to animals and residing in rural areas to document zoonotic exposure to *Brucella* sp., *Leptospira* sp., and hantaviruses amongst high-risk occupational groups.

### 2.2. Study area

**Figure 1:**
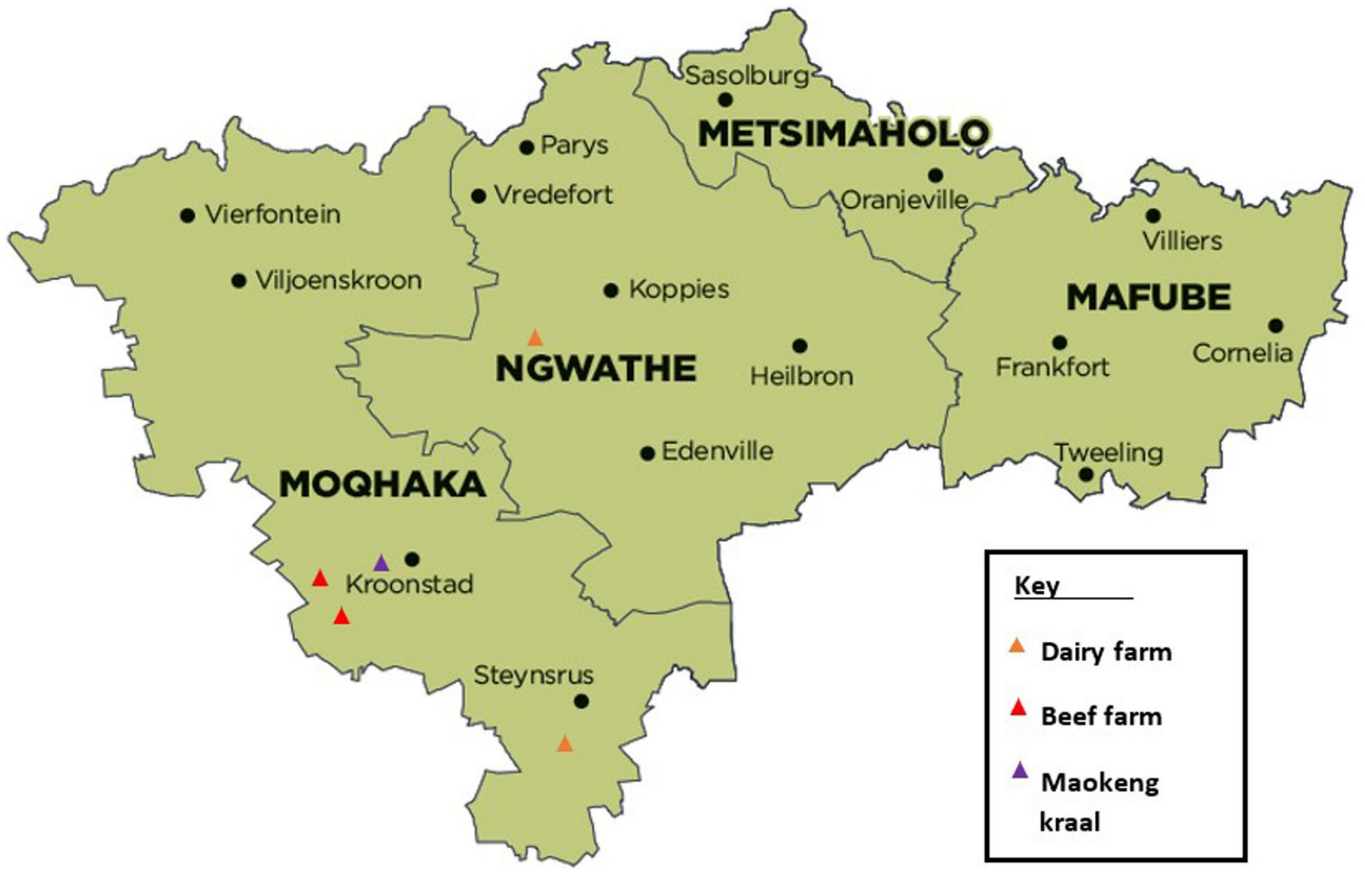
Study sites in the different municipal regions within the Fezile Dabi district, Free State province, South Africa. Available at https://municipalities.co.za/map/107.

This bTB and BB study was conducted on two distinct farming populations, one communal farm and four commercial farms consisting of two beef and two dairy farms. The informal communal farm was located in Maokeng, Kroonstad rural, Free State province, South Africa, and the commercial farms were located within the Moqhaka and Ngwathe municipal regions in the Free State province (Figure 1).

### 2.3. Study populations

#### 2.3.1. Animals

Two commercial dairy farms, designated as farms A and B and two commercial beef farms, designated as farms C and D, were selected by the Kroonstad State Veterinary Services as per the routine screening schedule. Convenience sampling was done for bTB and brucellosis based on the cattle availability on the farm on the day of testing and DALRRD testing history. The screening was conducted on all animals on each farm above the ages of 6 months for bTB and 18 months for BB.

#### 2.3.2. High-risk human occupational workers

The bTB and BB study comprised two sample groups, A and B. In group A, 26 on-site/prospective sputum and serum samples were collected from farmworkers in the Maokeng community kraal (*n* = 13), commercial dairy farm B (*n* = 7), and commercial beef farm C (*n* = 6). A convenience sampling method was used. All participants above 18 years old were approached and enrolled if they agreed to participate in the current study. Specimen collection could not be achieved on farms A and D due to COVID-19 travel restrictions.

In group B, a total of 301 archived serum samples collected from healthy individuals between April 2016 and February 2017 as part of a previous study (HSREC34/2016 and ETOVS152/06) were included. The individuals lived in rural areas and had exposure to animals due to their occupation.

All 327 samples (from groups A and B) used in this study were collected within the Free State province and included the following high-risk populations: farmworkers (*n* = 28), abattoir workers (*n* = 207), veterinarians (*n* = 12), stable grooms (*n* = 32), recreational hunters (*n* = 46), and laboratory workers (*n* = 2).

### 2.4. Tuberculosis test in animals

A total of 321 cattle were tested for bTB, including 33, 126, and 91 cattle from farms A, B, and C, respectively. Farm D was only scheduled for BB screening and not bTB (previously tested negative), and 71 cattle were screened for bTB from the Maokeng community kraal.

#### 2.4.1. Single intradermal skin test

All cattle herds from the Maokeng community kraal, farms A and C were initially screened for bTB using a single intradermal skin test (SIST), as described in the Bovine Tuberculosis Manual, approved by DALRRD (Department of Agriculture, Forestry and Fisheries of SA (DAFF), 2016). Briefly, the skin thickness of the animal was measured pre-injection using a Hauptner pistol grip. The animal was then injected intradermal with 0.1 ml of 5,000 International Units (IU) of bovine tuberculin purified protein derivative (PPD) (Onderstepoort Biological Products (OBP), Pretoria) using a McClintock syringe. After 72 h, reaction sites were observed for evidence of swelling or a color change and examined for reaction consistency (hard or soft swelling), presence of edema, and heat. The measurements were recorded, and the difference in skin thickness, pre-and post-injection, was determined for each animal by subtracting the measurement obtained after 72 h from the initial skin thickness measurement to determine reaction type.

Herds were regarded as negative when animals had a change in skin thickness of <6 mm, including non–specific reactions. Suspect herds were defined as having a single animal with an increase in skin thickness of >6 mm, combined with evidence of positive skin reactions. Herds with animals showing large typical inflammatory reactions with an increase of skin thickness of ≥20 mm were regarded as positive.

#### 2.4.2. Comparative intradermal skin test

The comparative intradermal skin test (CIST) was performed only on farms A and B due to COVID-19 restrictions. On farm A, all cattle were tested, whereas, on farm B, all SIST-suspect and -positive herds were re-tested after 3 months using a CIST. The CIST followed the same procedure as the SIST. However, instead of solely injecting 0.1 ml of 5,000 IU bovine tuberculin PPD, 0.1 ml of 2,500 IU avian tuberculin PPD (OBP, Pretoria) was injected approximately 20 cm apart. The bovine reaction increase was determined by subtracting the 72 h post-injection skin measurement (bovine PPD) from the pre-injection normal skin measurement. The avian reaction increase was determined by subtracting the 72 h post-injection skin measurement (avian PPD) from the pre-injection normal skin measurement. A positive difference between bovine and avian of <2 mm was regarded as a negative reactor; 3–4 mm suspect; an increase of >5 mm was regarded as a positive bovine reactor.

#### 2.4.3. Confirmatory interferon-gamma release assay

The interferon-gamma (IFN-γ) release assay was performed on heparinized blood samples collected from CIST-suspect and -positive reactors as per the Bovigam™ (Thermo Fisher Scientific, United States) standard operating procedure at the Tuberculosis Laboratory at Onderstepoort Veterinary Institute (OVI), within 6 h after collection. Briefly, blood samples were stimulated with bovine PPD, avian PPD, fortuitum PPD (OBP, Pretoria), and pokeweed mitogen (OBP, Pretoria) positive control and incubated at ±37°C for 24 h. Plasma was harvested, and interferon-gamma release was detected per the manufacturer’s instructions (Bovigam™).

The release of IFN-γ from stimulated blood was detected using a BovigamTM test kit per the manufacturer’s instructions. Steps requiring plate washing were done using a 96-well plate washer (BioTek ELx50, United States), and the optical density (O.D.) of the samples was measured at 450 nm using a plate reader (BioTek Elx800, United States). Whole blood stimulated with pokeweed mitogen was used as a positive control, and unstimulated blood was used as a negative control. The O.D. values for the plasma stimulated with bovine PPD, avian PPD, fortuitum PPD, and pokeweed mitogen were recorded as O.D.bov, O.D.av., O.D.fort, and O.D.pwh, respectively. Unstimulated blood was recorded as O.D.neg. Animals were considered bTB positive when (O.D.bov - O.D.av. >2 and O.D.fort - O.D.neg ≤0.15). Animals were classified as avian reactors when O.D.av. > (O.D.bov + 0.1 × O.D.bov). Animals demonstrating an immune response to bovine PPD and fortuitum PPD were classified as multiple reactors if (O.D.bov – O.D.av. <0.2 and O.D.fort – O.D.neg >0.15). Animals demonstrating an equivalent immune response to both bovine PPD and avian PPD were classified as equal reactors (O.D.bov + 0.1 × O.D.bov) > O.D.av. > (O.D.bov – 0.1 × O.D.bov). The test was considered valid if the O.D. value of the blood stimulated with pokeweed mitogen (O.D.pwh) was >0.5.

All samples stimulated with bovine PPD were initially screened to determine any positive reactors. Any sample with an O.D. of ≥0.38 was regarded as positive, as Michel (2008) described. All positive reactors were subject to re-testing with the inclusion of avian PPD, fortuitum PPD, and controls.

#### 2.4.4. Molecular characterization

From DALRRD’s biobank, previous samples were obtained from bTB-positive cattle from the same study farms and were characterized using Mycobacterial interspersed repetitive-unit-variable number tandem repeat (MIRU-VNTR). The typing was performed using the 24 MIRU-VNTR typing kit supplied by Quadruplex versions (GenoScreen, France), according to the manufacturer’s guidelines by the Tuberculosis Laboratory at OVI, Pretoria, South Africa. The MIRU-VNTR profiles were reported as numbers corresponding to the number of alleles at each locus and were entered in an excel sheet. These numerical patterns were then analyzed using the MIRU-VNTRplus database.^1^

### 2.5. Tuberculosis test in animal products

#### 2.5.1. Milk culture

Milk was collected from all CIST-suspected and positive female animals. All samples were transported and processed for culture at the Tuberculosis Laboratory at OVI, Pretoria, South Africa. Milk samples were decontaminated using 1% cetylpyridinium chloride to achieve a final volume of 150 mL and incubated at 20°C ± 2°C for 1 week in the dark. After that, samples were centrifuged at 3500 ×*g* for 30 min, the supernatant discarded, and the remaining pellet was inoculated onto 4X Lowenstein Jensen (LJ) - pyruvate and 2X LJ- glycerol media. Incubation followed at 37°C ± 2°C for 10 weeks.

### 2.6. Tuberculosis test in humans

#### 2.6.1. Sputum decontamination and culture

The 26 sputum samples collected were decontaminated using a BD BBL™ MycoPrep™ kit per the manufacturer’s instructions. Following this, a BD BACTEC™ MGIT™ 960 Supplement Kit was required for selective growth, containing both a growth supplement and an antibiotic mixture. Samples were cultured within a BD BACTEC™ MGIT™ 960 Mycobacteria Culture System at ±37°C for up to 42 days. Positive cultures were initially screened using a modified Ziehl–Neelsen staining technique to determine phenotypic characteristics.

#### 2.6.2. DNA extraction and line probe assay

DNA was extracted from positive cultures using a GenoLyse® DNA Extraction kit from Hain Lifescience according to instructions.

Per the manufacturer’s instructions, *M. tuberculosis* was amplified and genotyped using a GenoType Mycobacterium CM kit. Amplicon hybridization was performed using a GenoType Mycobacterium CM VER 2.0 kit (Hain Lifescience) in an automated GT–Blot 48 (Hain Lifescience) hybridization apparatus. Visible hybridization bands on the DNA strips were compared to a reference key to differentiate and speciate between the *M. tuberculosis* complex and 27 clinically relevant Nontuberculous Mycobacteria (NTM).

### 2.7. Brucellosis test in animals

A total of 1862 whole blood samples from the communal farm and commercial farms were collected from the tail vein of cattle. This included 33, 117, 449, and 1194 cattle from commercial farms A, B, C, and D, respectively. In the Maokeng community, kraal 69 cattle were screened for BB. These samples were sent to OVI or Grahamstown Veterinary Laboratory for brucellosis screening. An initial rose Bengal test (RBT) was performed on all samples, and confirmatory testing was performed on positive reactors using a complement fixation test (CFT).

### 2.8. Serological testing of human samples for antibodies against *Brucella* sp., *Leptospira* sp., and hantaviruses

All serum samples were screened for brucella IgG-specific antibodies using a commercially available indirect ELISA (Vircell; Granada, Spain), and steps were carried out per the manufacturer’s instructions. Optical density was measured at a wavelength of 450 nm (with a reference read at 630 nm) using a BioTek® 800TS™ Absorbance Reader (Winooski, United States). The mean O.D. value was calculated for the cut-off serum provided, and the antibody index was calculated as follows: (sample O.D./cut-off serum mean O.D.) × 10. An index of <9 was considered negative, 9–11 equivocal, and >11 positive. All results that returned as equivocal were re-tested.

*Leptospira*-specific antibodies were detected using a commercially available Panbio IgM ELISA (Windsor, Australia), according to the manufacturer’s instructions. Optical density values were measured at a 450 nm wavelength with a reference filter at 630 nm. The cut-off value was determined by calculating the average absorbance of the calibrators tested in triplicate, multiplied by the calibrator factor (batch specific). Results were calculated as “Panbio units”: sample absorbance/cut-off value. A result of <0.9, 0.9 to 1.1, and >1.1 was defined as negative, equivocal, or positive, respectively. All samples with an equivocal result were re-tested.

A commercially available EUROIMMUN Anti-Hanta Virus Pool 1 “Eurasia” ELISA (Lübeck, Germany) was used to detect hantavirus-specific IgG antibodies. This *in vitro* assay can detect human IgG antibodies against old-world hantavirus strains (Hantaan, Dobrava, and Puumala), and the procedure was carried out according to the manufacturer’s instructions. Results were determined semi-quantitatively. The ratio of the test sample to the provided calibrator was determined as follows: absorbance of the serum sample/absorbance of calibrator two (20 RU/ml). A ratio of <0.8 was considered negative, ≥0.8 to <1.1 equivocal, and ≥1.1 positive. Equivocal samples were re-tested.

### 2.9. Occupational and environmental zoonotic risk factors

Demographical, occupational information, food preparation practices, and risk factors (e.g., livestock exposure, source of livestock food products, and any reports of illness after a participant was directly exposed to animal tissue/fluids) were obtained through a questionnaire previously used (Vawda et al., 2018).

### 2.10. Statistical analysis

Database establishment and the necessary manipulation of data were done in Excel® 2016. Due to skewed distributions, descriptive statistics were calculated, namely frequencies, percentages for categorical variables, medians, and quartiles for numerical variables. Associations between categorical variables and laboratory outcomes were assessed using chi-squared or Fisher’s exact test in the case of sparse data. Differences between laboratory outcome groups regarding numerical variables were assessed using Mann–Whitney tests. All statistical analyses were performed by the University of the Free State Department of Biostatistics using SAS Version 9.4.

### 2.11. Ethical considerations

Ethical approval was obtained from the University of the Free Health Sciences Research Ethics Committee (UFS-HSD2019/1075/270801), Animal Research Ethics Committee (UFS-AED2019/0111), and Environmental & Biosafety Research Ethics Committee (UFS-ESD2019/0086). Furthermore, permission was obtained from the Department of Agriculture, Land Reform and Rural Development (DALRRD) before any animal testing was conducted. Verbal consent from farm owners was obtained for the collection of animal samples. Signed informed consent was obtained from volunteers before the samples were collected.

## 3. Results

### 3.1. Tuberculosis test in animals

#### 3.1.1. Single and comparative intradermal skin test

A total of 321 cattle were screened for bTB using SIST and CIST, including 71/321 (22.1%) from the Maokeng community kraal, 33/321 (10.3%) from farm A, 126/321 (39.3%) from farm B and 91/321 (28.3%) from farm C. bTB results were read 72 h after inoculation. bTB results were available for 301/321 (93.8%). The Maokeng community kraal had the least number of animals that returned for bTB results (*n* = 51/71). Based on the SIST results, 3/51 (5.9%) cattle were suspect reactors in the Maokeng community, and 4/33 (12.1%) in farm A (Table 1). No positive bTB results with ≥20 were detected.

**Table 1:** Results of the single intradermal skin test and comparative intradermal skin test in cattle from the Moqhaka and Ngwathe municipality regions.

		Community	Commercial dairy		Commercial beef
		Maokeng	Farm A	Farm B	Farm C
No. of cattle screened with SIST		71	33	N/A	91
No. of cattle returned after 72h for result readings		51	33	N/A	91
SIST screening results	Positive	0	0	N/A	0
	Suspect	3	4	N/A	0
	Negative	48	29	N/A	91
No. of cattle screened with CIST		N/A	33	126	N/A
No. of cattle returned after 72h for result readings		N/A	33	126	N/A
CIST screening results	Positive	N/A	0	8	N/A
	Negative	N/A	33	110	N/A
	Suspect	N/A	0	8	N/A

*Farm D was not scheduled for bTB screening. SIST, single intradermal skin test; CIST, comparative intradermal skin test. N/A represents not applicable as testing was not done.

Based on the bTB confirmatory test, CIST, the four SIST-suspected positive animals from farm A were negative using CIST. In contrast, CIST results indicated that 8/126 (6.3%) cattle were positive reactors and 8/126 (6.3%) were suspect reactors on farm B. No CIST was performed on samples from animals in the Maokeng community due to the animals not being available for the three-month follow-up due to covid restrictions.

**Figure 2:**
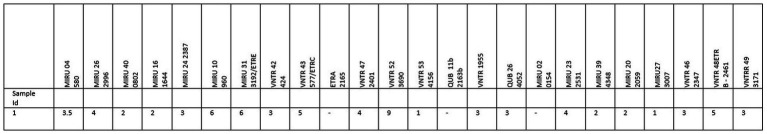
Mycobacterial interspersed repetitive-unit-variable number tandem repeat (MIRU-VNTR) typing of *Mycobacterium tuberculosis* isolated from a previously bTB CIST-positive cattle from farm B.

The IFN-γ release assay was scheduled to be performed on all 16 CIST-suspect and -positive animals from farm B. Of the 16 positive/suspect animals, whole blood was collected from 13/16 (81%). Two animals died during the three-month waiting period, and the third had hypocalcemia when sampling was conducted (no sample was available). Therefore, the IFN-γ assay was performed on 7/8 CIST-positive and 6/8 -suspect animals. Three of the 13 cattle had a positive IFN-γ result, two bovine reactors, and one avian reactor. The remaining ten samples were negative (Table 2). Subsequently, all milk samples collected were culture-negative (Figure 2).

**Table 2:** Interferon-gamma release assay and milk culture result from cattle with positive and suspect CIST reactions.

Animal status based on CIST result	CIST skin reaction increase (mm)	External characteristics of injection site	IFN-γ results	Milk culture results
Positive	10.4	A, skin condition	Positive	Negative
Positive	6.7	C	Negative	Negative
Positive	5.6	C	Negative	Negative
Positive	5.4	C	Negative	Negative
Positive	4.8	C, mild D	Positive	Negative
Positive	4.7	C	Negative	Negative
Positive	4.6	C	Negative	Negative
Suspect	3.9	C	Negative	Negative
Suspect	3.9	C	AV	Negative
Suspect	3.8	C	Negative	Negative
Suspect	3.7	F	Negative	Negative
Suspect	3.6	C	Negative	Negative
Suspect	3.3	C	Negative	Negative

C, circumscribed; A, adhesions, D, diffuse; F, flat; AV, avian reactor.

#### 3.1.2. Confirmatory gamma-interferon assay

##### 3.1.2.1. Molecular characterization

###### 3.1.2.1.1. Tuberculosis test in humans

*Mycobacterium bovis* or MTBC species were not detected in the 26 human sputum samples that were tested. Seven of the 26 samples (27%) were flagged as culture positive. *Nocardia* sp. was detected in 2/7 (28.6%) samples, *Mycobacterium intracellulare* in 2/7 (28.6%) samples, and 3/7 (42.8%) samples were identified as other *Mycobacterium* sp. excluding MTBC and the 27 clinically significant NTMs (Table 3).

**Table 3:** Sputum culture-positive results collected from farm workers and herd status.

Sample no.	Sex	Age	Herd status	Species
**Maokeng community kraal**				
1	Male	52	Suspect	*Nocardia* sp.
2	Male	23	Suspect	*Mycobacterium intracellulare*
3	Male	74	Suspect	*Mycobacterium intracellulare*
5	Female	57	Suspect	*Mycobacterium* sp.
**Commercial dairy farm B**				
23	Female	30	Positive	*Mycobacterium* sp.
**Commercial beef farm C**				
21	Male	27	Negative	*Mycobacterium* sp.
24	Male	25	Negative	*Nocardia* sp.

### 3.2. *Brucella* test in animals

A total of 1862 cattle were available for *Brucella* sp. using the RBT (Table 4). However, three samples were hemolysed and not included. A total of 52/1859 (2.8%) cattle had a positive RBT result and were subjected to confirmatory testing using the CFT. From these results, 19/52 (36.5%), 6/52 (11.5%), and 27/52 (51.9%) were confirmed CFT positive, suspect, and negative, respectively.

**Table 4:** Livestock rose bengal test, complement fixation test, and bovine brucellosis results of cattle from Maokeng community kraal and four commercial farms linked to farmworkers IgG results.

	No. of conclusive results	No. of RBT positive reactions (%)	No. of CFT positive reactions (%)	No. of CFT suspect reactions (%)	No. of CFT negative reactions (%)	No. of farmworkers with a positive IgG detected/No. tested (%)
**Community**						
Maokeng Com Kraals	69	8 (11.6)	6 (8.7)	0	2 (2.9)	3/13 (23.07)
**Commercial (dairy)**						
Farm A	33	0	N/A	N/A	N/A	N/A
Farm B	117	0	N/A	N/A	N/A	1/13 (7.69)
**Commercial (beef)**						
Farm C	448	9 (2)	0	4 (0.9)	5 (1.1)	N/A
Farm D	1192	35 (2.9)	13 (1.1)	2 (0.2)	20 (1.7)	N/A
Total	**1 859**	**52 (2.8)**	**19 (1)**	**6 (0.3)**	**27 (1.5)**	

N/A represents not applicable as testing was not done.

### 3.3. Serological testing of human samples for antibodies against *Brucella* sp., *Leptospira* sp., and hantaviruses

**Figure 3:**
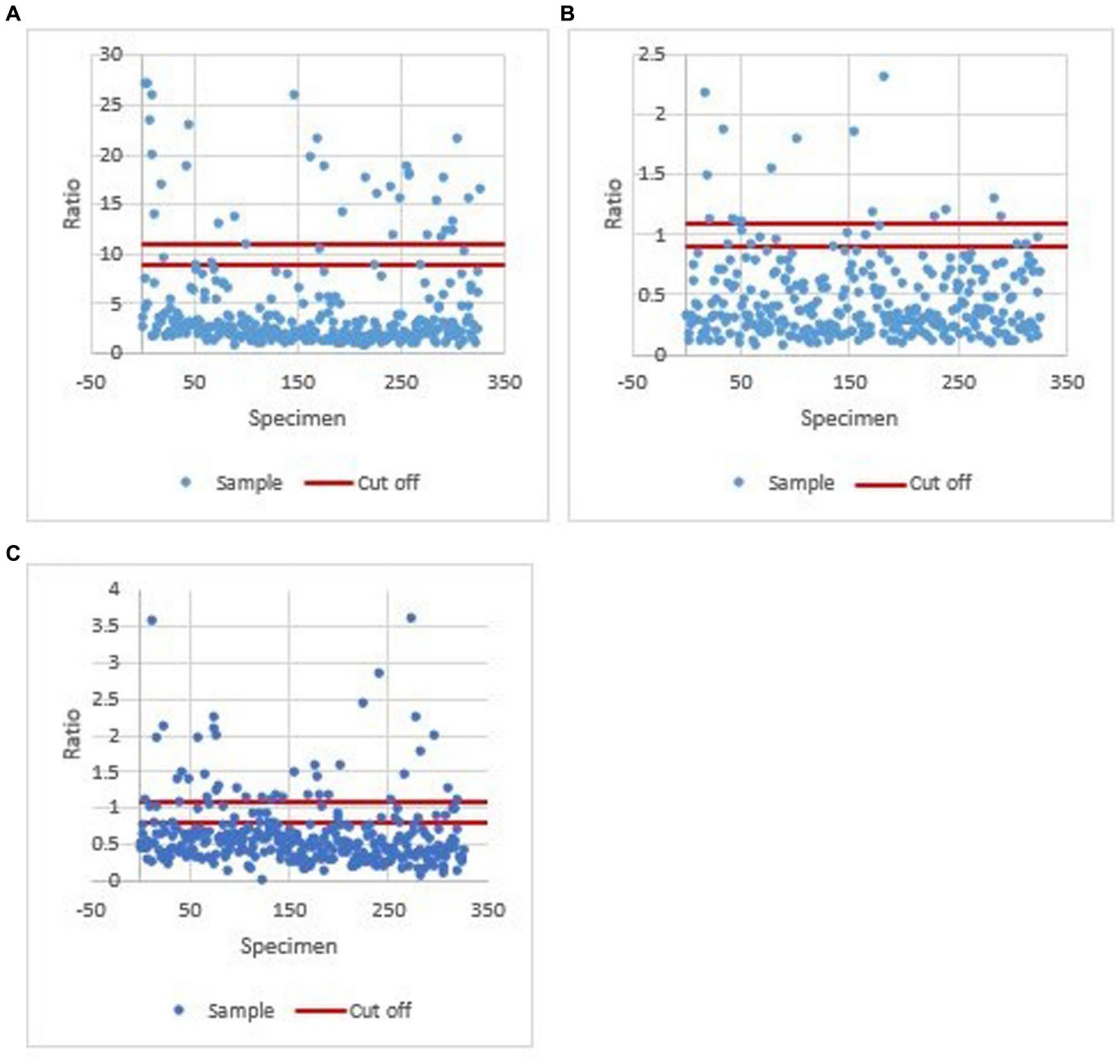
Ratio values calculated for each sample in all three assays (*Brucella* sp. *Leptospira* sp., and hantavirus). **(A)** Ratio values calculated for each sample in the *Brucella* sp. IgG ELISA assay. A ratio of <9 was considered negative, 9–11 equivocal, and > 11 positive. **(B)** Ratio values calculated for each sample in the *Leptospira* sp. IgM ELISA assay. A ratio of <0.9, 0.9 to 1.1, and >1.1 was defined as negative, equivocal, or positive, respectively. **(C)** Ratio values calculated for each sample in the hantavirus IgG ELISA assay. A value of <0.8 was negative, ≥0.8 to <1.1 equivocal, and ≥1.1 positive.

A total of 327 human serum samples were screened for IgG antibodies against *Brucella* sp. and hantaviruses and IgM antibodies against *Leptospira* sp. For each assay, the results were normalized by calculating the ratio for each sample to that of the cut-off control (according to manufacturers’ instructions). Ratio values calculated for each sample in all three assays (*Brucella* sp., *Leptospira* sp., and hantavirus) were plotted (Figures 3A–C).

Results obtained from the *Brucella* sp. IgG, *Leptospira* sp. IgM and hantavirus IgG ELISA for each occupational group are shown in Table 5. *Brucella* IgG-positive reactors were detected in all occupations. Specific to group A (the 26 farm workers), *Brucella* IgG antibodies were detected in three Maokeng community farmers and one farm B worker (Table 3). *Leptospira* sp. IgM and Hantavirus IgG antibodies were mainly detected in abattoir workers and stable groomsmen.

**Table 5:** Number of positive *Brucella* sp. IgG, *Leptospira* sp. IgM and hantavirus IgG reactors per occupation group.

Occupation	Number of positive samples, ratio of >11 or >1.1* (%)			
	Number of participants (%)	*Brucella* sp. IgG reactors (%)	*Leptospira* sp. IgM reactors (%)	Hantavirus IgG reactors (%)
Abattoir workers	207 (63.3)	20 (9.7)	14 (6.7)	26 (12.6)
Veterinarians	12 (3.7)	6 (50.0)	0 (0)	1 (8.3)
Stable groom	32 (9.8)	2 (6.3)	2 (6.3)	4 (12.5)
Recreational hunters	46 (14.1)	2 (4.3)	1 (2.2)	4 (8.7)
Farm workers	28 (8.6)	4 (14.3)	0 (0)	3 (10.7)
Laboratory workers	2 (0.6)	1 (50.0)	0	0 (0)
Positive rate				26 (68.4)
Total (%)	327	35 (10.7)	17 (5.2)	38 (11.6)

*Depending on manufacturers’ instructions.

### 3.4. Occupational and environmental zoonotic risk factors

*P*-value analysis of the *Brucella* IgG positive results showed that age (value of *p* = 0.0008), veterinary work (value of *p* = 0.0006), and laboratory work (value of *p* = 0.031) were all significant risk factors. Based on the participant’s response to the questionnaire, illness post-exposure to animal tissue/blood (value of *p* = 0.029) was statistical significance to *Brucella* IgG seropositivity.

The maximum, minimum, and mean age for all Hantavirus IgG-positive participants was 65, 19, and 39, respectively. Statistical analysis of Hantavirus IgG showed no significance (value of *p* < 0.05) to any risk factor variables.

Analysis conducted on the *Leptospira* seropositive IgM results depicted abattoir work or informal slaughtering (value of *p* = 0.024) as the only significant risk factors.

## 4. Discussion

This study aimed to investigate the prevalence of *M. bovis* and *Brucella* sp. in cattle and farm workers in two farming communities (communal and commercial) and their associated risk factors. In addition, this study documents occupational and environmental exposure to *Brucella* sp., *Leptospira* sp., and hantaviruses across the Free State province, South Africa.

Based on the available data and confirmatory IFN-γ assay and skin tests, the cattle bTB prevalence detected in this study was 0.7% (two animals), all originating from a commercial dairy farm B (Table 1). These findings are lower than reports from other sub-Saharan countries, 6.2% in Algeria, 7.4% in Sudan, and ± 27% in Ethiopia (Ameni et al., 2011). Our results demonstrate the potentially effective control schemes in lowering bTB transmission in the study site. In South Africa, the bTB eradication and control scheme was implemented in 1969, following the ‘test and slaughter’ approach, due to the economic importance of the disease (Michel et al., 2006). The approach has been met with great success and led to a substantial decrease in bTB in cattle within the commercial sector, from a prevalence of 11.8% in 1971 to 0.39% in 1995 (Arnot and Michel, 2020). Another explanation for the lower incidence rate could be an absent reservoir host, such as buffalo or other wildlife species neighboring the study area. Spill-over of bTB from wildlife to neighboring livestock does reportedly occur at the wildlife-livestock interface in South Africa (Musoke et al., 2015).

Unfortunately, neither positive animals were slaughtered to inspect for visible lesions and culture. Therefore, no differentiation could be made between *M. bovis* and *M. tuberculosis* or any other member of the MTBC. However, on farm B, in 2018, an animal had a positive CIST result and was subsequently slaughtered. No visible lesions were detected (personal communication from the veterinarian). Nonetheless, lymph node tissue was sent for culture, and *M. tuberculosis* was confirmed (Figure 2). Therefore, the possibility arises that both animals may be infected with *M. tuberculosis* based on the farms’ history. Both animals were first-time reactors, having tested negative with the CIST 8 months before the positive result.

Conversations with the farm owner and workers revealed that in 2018 (when *M. tuberculosis* was cultured from an animal on the farm), a farm worker diagnosed with TB was present. The worker passed away at the end of 2018. Further investigations into the possibility of reverse zoonotic TB are required on farm B. Additionally, throughout this study, no cattle were introduced into the herd. Therefore, another possible explanation could be latent TB reactivation. Previous reports have shown that cavitation of caseous lesions can occur in cattle herds infected with bTB and is required for the bacteria to go into a state of dormancy (Van Rhijn et al., 2008). This phenomenon of reactivation is documented more frequently in humans than in cattle. However, the Australian TB eradication program reports evidence of latent bTB reactivation in cattle whereby several infected animals were detected, culled, and then years after, more infected animals were detected with no external infection source (Cassidy, 2006).

A possible zoonotic transmission source to humans was not found in commercial or community-based settings, as all sputum culture results were MTBC negative. Rather symbiotically similar *Nocardia* sp. was isolated from two farm workers (2/7), who responded in the questionnaire that they regularly consume unpasteurized milk. Based on the SIST results, one of the *Nocardia* sp. positive cases was from a farmer in the Maokeng community with a bTB suspect herd. Previous studies have confirmed the transmission of *Nocardia* sp. from cattle to humans by consuming dairy products from cattle infected with the bacteria (Wahba et al., 2011).

Limited studies have reported BB’s prevalence or incidence rate in South Africa cattle populations. Previous findings have reported on the seroprevalence of BB in Gauteng, Mpumalanga (Mnisi area), and KwaZulu Natal and determined seroprevalence of 2.33, 0.88, and 1.3%, respectively, with the latter two focusing on communal cattle in municipal dip tanks (Matekwe, 2011; Chisi et al., 2014; Govindasamy et al., 2021). Moreover, introducing compulsory calf vaccinations in South Africa has considerably decreased the overall BB prevalence from approximately 10.5% in 1976 to 1.4% in 1988 (Godfroid et al., 2004). These results agree with the findings from this study, where an incidence rate of 1% was determined. Based on the confirmatory BB test and CFT assay, 1% of all cattle screened were positive. Of this, 6/69 (8.7%) cattle were CFT-positive from the Maokeng community, whereas commercial beef farm D had 13/1192 (1.1%) BB-positive animals. The BB higher seroprevalence in the Maokeng farm was expected as literature reports that in subsistence farming communities, BB almost always exceeds 5% in sub-Saharan Africa (Chisi et al., 2014). Bovine brucellosis’ higher incidence rate in communal settings is likely attributed to how animals are managed and a lack of disease awareness among farmers (Cloete et al., 2019). In commercial settings, animals are raised on enclosed land where movement is restricted and controlled. In addition, in these settings, BB control measures such as mass herd vaccinations and annual testing are implemented more stringently to adhere to specific standards. However, in communal farming, grazing land is shared amongst farmers where cattle herds interact with other herds, increasing the risk of transmission (Madzingira et al., 2020).

*Brucella* sp. IgG antibody was detected in 4/26 (15.4%) farm workers, including 3/13 (23%) Maokeng community farmers and one farm B worker. The positive reactors from the Maokeng community kraal were from a farm on which BB-positive herds were identified. Two of the three farmers reported consuming unpasteurized milk regularly from their herd, and the third confirmed to have assisted his animals in parturition several times over the past years. The positive reactor from the commercial beef farm was from a BB suspect herd, as determined by the CFT results. The incidence rate of *Brucella* sp. seropositivity in the community could result from limited knowledge regarding disease prevention and transmission and the higher incidence among the herd, increasing the risk of potential exposure (Cloete et al., 2019). At the time of specimen collection, all participants appeared to be healthy. However, *Brucella* sp. can cause persistent chronic infections in humans, and a clinical form of the disease may develop due to the individual being immunocompromised (Ulu-Kilic et al., 2013). Participants were made aware of the results and advised to visit their healthcare facilities in the event they feel unwell.

A few studies have been conducted on occupational exposure to *Brucella* sp. amongst healthy individuals in South Africa. These include a survey study of zoonotic diseases contracted by 88 South Africa veterinarians (Gummow, 2003). Out of the 88 veterinarians surveyed, 56 (63.6%) contracted one or more zoonotic diseases, with 7/88 (8%) reporting illness due to brucellosis. In a study conducted on 64 dip-tank workers (people who work at dip tanks) in Bushbuckridge, Mpumalanga, an incidence rate of 0% (0/64) was determined using a Brucellacapt® assay with a reported sensitivity and specificity of 96 and 97.5%, respectively (Simpson et al., 2018). The higher seroprevalence obtained in this study might indicate a higher disease burden in the Free State province. The higher seropositivity is likely due to the participants’ occupation and recreational activities, putting them at a higher risk of contracting an infection. Based on the results of this study, two high-risk occupational groups were identified as having a higher *Brucella* IgG seropositive rate compared to the other occupations: laboratory workers (*p* = 0.031) and veterinarians (*p* = 0.0006; Table 5). However, these findings were based on only two samples, and further research is required.

In South Africa, there are sporadic cases of leptospirosis reported by the National Institute for Communicable diseases (NICD). This report identified abattoir workers as a high-risk occupational group for *Leptospira* sp. in the Free State, indicating that *Leptospira* sp. are circulating within the Free State province. The populations screened had possible exposure from various sources, including horses, livestock, and rodent populations as rural residents, hence although not possible to identify the source of infection, the results justify additional investigations to determine the prevalence of *Leptospira* sp. in livestock, domestic animals, rodents and wildlife and to identify the serovars circulating for diagnostic purposes. Abattoirs should also enforce more strenuous preventative measures to reduce infections.

Hantaviruses are usually transmitted from environmental exposure, and the presence of hantaviruses in South Africa has, to date, not been confirmed. The hantavirus IgG seroprevalence data are similar to data obtained in other studies (Klempa et al., 2013, Witkowski et al., 2014). Although no conclusions can be drawn from this limited study in the absence of confirmatory assays such as neutralization tests, more extensive serosurveillance studies are justified to provide more information regarding the presence of hantaviruses in the country. Hantaviruses have not previously been associated with disease in Africa however, medically significant rodent borne hantaviruses belonging to various genera circulate in Asia, Europe and North and South America. The presence of potential rodent hosts in Africa suggest that they are likely to occur and hence warrant investigation as a potential zoonotic pathogen among at risk populations.

In conclusion, South Africa has a large proportion of the human population dependent on animals for their livelihood, whether as a source of food, trade, companionship, or services, and the importance of a One Health approach to zoonotic pathogens should be encouraged. Therefore, more emphasis should be placed on populations at higher risk of contracting zoonotic infections regarding epidemiological investigations. Identifying high-risk populations for different zoonotic diseases across other geographical regions will ultimately aid in implementing effective preventative measures and assist clinicians in diagnosing undifferentiated febrile illness patients.

## Data availability statement

The raw data supporting the conclusions of this article will be made available by the authors, without undue reservation.

## Author contributions

CW, FB, and JM created the study concept and design. CW, FB, NH, WZ, TA, and JM performed data collection and analysis. NH drafted the first manuscript. All authors contributed to the article and approved the submitted version.

## Funding

This work was supported by the Department of Science and Technology, National Research Foundation (NRF) Thuthuka research grant [UID107432 to JM], South African Research Chairs Initiative (SARChI) Vector-borne and zoonotic pathogens research [U98346 to FB].

## Conflict of interest

The authors declare that the research was conducted in the absence of any commercial or financial relationships that could be construed as a potential conflict of interest.

## Publisher’s note

All claims expressed in this article are solely those of the authors and do not necessarily represent those of their affiliated organizations, or those of the publisher, the editors and the reviewers. Any product that may be evaluated in this article, or claim that may be made by its manufacturer, is not guaranteed or endorsed by the publisher.

## Footnotes

^1^ www.miru-vntrplus.org
